# Use of complementary and alternative medicine by cancer patients at the University of Nigeria Teaching Hospital, Enugu, Nigeria

**DOI:** 10.1186/1472-6882-7-28

**Published:** 2007-09-12

**Authors:** Emmanuel R Ezeome, Agnes N Anarado

**Affiliations:** 1 Department of Surgery, University of Nigeria Teaching Hospital, Enugu Nigeria; 2 Department of Nursing Sciences, College of Medicine, University of Nigeria, Enugu Campus, Nigeria

## Abstract

**Background:**

The use of Complementary and Alternative Medicine (CAM) by cancer patients is very common and varies between populations. The referenced English literature has no local study from Africa on this subject. This study was conducted to define the prevalence, pattern of use, and factors influencing the use of CAM by cancer patients at the University of Nigeria Teaching Hospital Enugu (UNTH-E), Nigeria

**Method:**

Face-to-face interviews using semi-structured questionnaire were used to determine the use of CAM by cancer patients. All consenting cancer patients were interviewed as they presented at the core surgical units of the UNTH- E, from June 2003 to September 2005.

**Results:**

160 patients were interviewed; 68 (42.5%) were males and 94 (57.5%) were females. Ages ranged from 13–86 years. Breast, urogenital system, gastrointestinal system, and soft tissue cancers predominated. One hundred and four patients (65.0%) have used CAM at some time during their current cancer illness; 56 (35.0%) patients have not used any form of CAM. There were more females than males among the non-CAM users. The use of CAM was not affected by age, marital status, level of education, religious affiliation, or socioeconomic status. The most frequently used CAMs were herbs (51.9%), faith/prayer healing (49.4%), aloe vera (23.1%), Forever Living Products (16.3%), medicinal tea (14.4%), and Blackstone (12.5%). Over 23% of those who used CAM were satisfied, but 68.3% were disappointed. Most users (67.3%) did not see any benefit from the CAM, but 25% could describe some specific benefits. More than 21% of users reported various unwanted effects. While 86.5% of CAM users will use orthodox medicine instead of CAM in the future, 9.6% will use the two together to help each other. Most users (79.8%) will not repeat CAM or recommend its use for cancer. The majority of patients (55.8%) did not mention their use of CAM to their doctors – mostly because the doctor did not ask.

**Conclusion:**

CAM use is common among cancer patients in Nigeria. Most users do not obtain the expected benefits, and adverse events are not uncommon. Every clinician in the field of oncology should ask his/her patients about the use of CAM; this knowledge will enable them to better counsel the patients.

## Background

The innate urge among human beings to try new and alternative ways of relieving suffering is exemplified by the popularity of complementary and alternative remedies during sickness [1-3]. This desire is more in areas in which conventional methods have failed to provide satisfactory solutions to diseases and ailments, such as in HIV infection and cancer [4-6]. The need to use Complementary and Alternative Medicines is also fueled by the quest for therapies considered more congruent to one's values, beliefs, and philosophical orientation towards health and life [1]. Complementary and Alternative Medicines (CAM) are defined as medical and health care systems, practices, and products that are not currently considered an integral part of conventional medicine [7]. Each particular therapy may be considered complementary if it is used in addition to conventional medical treatment; it is viewed as an alternative if the patient decides to use it in place of a prescribed medical treatment. Many studies done in western countries have documented that CAM use is both very common and varies among populations [2,3]. It is estimated that 30% to 50% of the general adult population of industrialized nations use one form of CAM or another [2]. Studies of cancer patients in the industrialized world documented a 7–83% prevalence rate for the use of CAM [5,6]. The type of CAM therapies vary, depending on age, level of income, level of education, and perceived cause and prognosis of the disease. The use of CAM in industrialized nations is more common among females; young adults/middle aged individuals, members of higher socioeconomic classes, and persons with higher levels of education [2,3,8].

Very few studies have described the use of CAM in third world countries. Only one study in the English literature has evaluated the prevalence of CAM in a general population. Singh, et al. reported a prevalence of 38.5% among the general population of Indians living in Chatsworth, South Africa [9]. Among the cancer populations, studies from the developing countries are mainly from Turkey and countries in Asia [5,10]. The prevalence of use of CAM among cancer patients in Turkey ranged from 54.5% to 61% [11,12]. A prevalence rate of 64% was recorded in advanced cancer patients in Taiwan [13]. These studies from developing countries suggest that perhaps those factors that have been known to facilitate the use of CAM in developed western countries, such as level of income and educational attainment, may indeed be working in the opposite direction in less developed countries. Data from indigenous African cancer populations have consistently been lacking in all the major reviews of use of CAM therapies and remedies in the world literature [5,10].

In Nigeria, the rate of CAM use among cancer patients is unknown. The use of traditional herbs and remedies are however well known and relatively common [14,15]. What biomedicine considers as CAM today has been a way of life for most Nigerians. Also, the cost of western medical treatment and inadequate penetration of the communities by western orthodox medicine makes CAM more appealing to the people. In addition, there are almost no medical malpractice litigations to limit and regulate the use of non orthodox remedies. Equally, cancer is considered a death sentence in Nigeria [15]. Its cause in many cases is attributed to non material causes beyond biomedicine so that western medicine is largely seen as ineffective in its treatment. It is therefore expected that the use of CAM in cancer patients will be commensurately higher than it is in western populations. We do not yet know which factors are most critical in influencing Nigerian cancer patients to use CAM. We also do not know what potential benefits or adverse outcomes can occur when Nigerian patients on conventional western medicine use standard therapy either concurrently or sequentially with CAM.

This study aims to define the prevalence, pattern of use, and factors influencing the use of CAM by cancer patients at the University of Nigeria, Enugu (UNTH-E), Nigeria.

## Methods

This was a cross-sectional study involving the direct administration of questionnaires to all cancer patients seen at the core surgical units of the UNTH-E, Nigeria from June 2003 to September 2005.

### The Questionnaire

The questionnaire was developed after an extensive literature search on CAM in cancer patients, as well as inquiries within the local community on forms of CAM being used. The initial questionnaire was refined and pre-tested with a focus group of five cancer patients in the surgical oncology unit of the hospital. The pilot patients were also asked to suggest CAMs that were not listed on the questionnaire. The final questionnaire was used to train 3 interviewers (nurses) who administered the questionnaires throughout the study. Interviewer training involved a 2-3-hour session on the processes of interviewing, rationale for the study, cancer treatment modalities, questionnaire items, and confidentiality. Each interviewer then administered the questionnaire to three patients. The interviewers were debriefed by one of the investigators (RE), and responses to the questions were cross checked for clarity and validity. A few of the question items were revised to improve clarity while some questions were removed for lack of relevance. This step also constituted the final validation of the questionnaire [see Additional file 1].

### Patient recruitment

All cancer patients managed in the surgical units of the UNTH-E, Nigeria from June 2003 to September 2005 were recruited into the study as long as they consented to give the required information. Ethical clearance was obtained from the Hospital's Institutional Review Board. Patients whose condition precluded the ability to give informed consent were excluded from the study. Patients were recruited consecutively as they presented at the clinics and wards. Information about the research was given verbally to each patient; those who gave consent were then interviewed. The first step in administering the questionnaire was the assurance section, in which the patients were informed that the information sought was not part of their treatment and would in no way influence the treatment of their cancer. The low level of education in our society necessitated a structured, face-to-face interview. The interviews took place on admission in the oncology clinic and in the wards. The patients were informed that they were free to decline answering any question with which they were not comfortable. Physicians who were in any way involved in the treatment of each patient were not present during the interview.

The questionnaire included demographic data, such as age, sex, marital status, socioeconomic status, and highest level of education attained. Questions were asked about the type of cancer, part of the body involved, previous treatments received, and the treatment that the patient was currently receiving. Information on the type of cancer and stage of the disease were obtained or cross checked from the case notes. Each patient was asked whether he/she had used any substance not recommended by the doctor to treat this cancer. Each patient was presented with a list of known CAM remedies. The patient was then asked whether he/she had used any of them before the diagnosis of this cancer, during this cancer, or planned to use them in the future. Each patient was also given an opportunity to mention other CAMs that were not listed, but which they had used during this illness. Patients who had used CAM at least once during the current cancer were regarded as CAM users; non-users had not used CAM at all during this cancer. Those who had used CAM before in their lives but not for this current illness were also considered non-users. CAM practices and therapies presented to the patients included: alternative medical systems (Chinese medicine, Indian medicine, acupuncture, homeopathy, ritual sacrifice, divination/incantations, specified folk remedies); mind-body interventions (massage, manual healing/therapeutic touch, mind-body technique, hypnosis, visualization/vision, meditation, and faith healing/prayer house healing), biologically-based treatments (herbal drugs, high dose/mega vitamins, Forever living products, aloe vera, GNLD products, medicinal tea, green tea, Kosagog tea, special diets/nutritional therapies, urine therapy, mineral therapy, animal extracts, python fat, shark cartilage, black stone, nutri water, and Tuja 1000), manipulative and body-based methods (local surgery/scarifications, bloodletting/coup, chiropractics, osteopathy/bone setting) and energy therapy (bioelectromagnetics, ozone/oxygen therapy).

CAM users were asked how frequently they used CAM, how they got the information about the CAM, what useful effect they were hoping to get from CAM, and how they had actually benefited from the CAM. They were asked if they had discontinued or hoped to discontinue CAM or conventional treatment, or whether they had to use CAM and orthodox treatment concurrently. The questionnaire also asked whether there were advantages to the CAM that the patient wished were available in conventional treatment. Finally, the patients were asked whether their doctor knew they were using or had used CAM, and whether they perceived any impediments to discussing their use of CAM freely with their doctor.

### Statistical analysis

The patients were classified into CAM users and non-users. The two groups were compared with respect to demographic characteristics and other factors that influence the use or non use of CAM in cancer. The data were analyzed using SPSS^® ^statistical software version 9.0. Chi square was used for comparison between the two groups, with the level of significance at p = 0.05.

## Results

A total of 199 patients with solid tumors were seen in the surgical section of the hospital during this period, 160 (80.4%) of them were interviewed in the study, the rest were too sick to participate, refused consent or were not approached. Sixty eight (42.5%) of participants were males, and 92 (57.5%) were females. Their ages ranged from 13–86 years, with a mean age of 52.3 years and a median age of 51.0 years. The majority of the patients have been on treatment for one month-two years. More than half of the patients (56.9%) have had surgery, 28.8% have received anti-cancer drugs, and 2.5% have had radiotherapy. At the time of the interview, 20.6% were on admission for surgery, 50.6% were receiving chemotherapy, and 3.8% were on symptomatic palliative therapy. The distribution of cancers represented the typical spectrum of patients we usually see in our oncology clinic and surgical wards: breast cancer (36.3%); urogenital tract cancers (19.4%); gastrointestinal tract cancers (16.9%); soft tissue tumors (7.5%). Other types included respiratory tract cancers (2.5%), head and neck cancers (1.9%), gynecological malignancies (1.9%), and lymphomas (0.6%).

When asked whether they have used anything other than what their doctors recommended in treating the cancer, 91 patients (56.9%) said they have and 65 patients (40.6%) said they have not. However, when each patient was presented with examples of individual CAMs, 104 (65.0%) of the patients said they have used at least one of them at some time during this cancer; 56 (35.0%) patients have not used any form of CAM at all.

Table 1 compares the demographic characteristics of CAM users and non users. More females were in the group of patients that have never used any form of CAM during the current illness (p = 0.052). While more of the "never married" patients have used CAM, a greater number of patients who were divorced, separated, or widowed were found to be non-CAM users; these differences were not statistically significant. Patients who had no formal education were more likely to have used CAM than those who had up to post-primary level of education or more. Again, the differences were not significant. There were more Catholics among the non-CAM users (67.8% vs. 57.7%), and disproportionately more of the Pentecostals were found among the CAM users (22.1% vs. 8.9%). These differences were also not significant. Other religious groups like Anglicans, Traditional religious adherents and Muslims were evenly distributed between the CAM users and non users.

**Table 1:** Demographic characteristics of CAM users and non users

**Parameter**		**CAM users (%)**	**Non CAM users (%)**	**significance**
Sex	Male	50 (48.1)	18 (32.1)	P = 0.052
	Female	54 (51.9)	38 (67.9)	
Mean age		51.9 yrs	53.0 yrs	P > 0.05
Marital status	Married	81 (77.9)	40 (71.4)	P = 0.050
	Not married	11 (10.6)	2 (3.6)	
	Widowed	12 (11.5)	11 (19.6)	
	Divorce/separated	-	2 (3.6)	
	No response	-	1 (1.8)	
Level of education				
	Non	14 (13.5)	5 (8.9)	P > 0.05
	Primary	26 (25.0)	14 (25.0)	
	Post primary	28 (26.9)	19 (33.9)	
	Tertiary	32 (30.8)	18(32.1)	
	No entry	4 (3.8)		
Socioeconomic status	Low income	49 (47.1)	24 (42.9)	P > 0.05
	Middle income	46 (44.2)	26 (46.4)	
	High income	5 (4.8)	1 (1.8)	
	No entry	4 (3.8)	5 (8.9)	

The most commonly used forms of CAM (Table 2) were the biological-based treatments, including herbs (51.9%), aloe vera (23.1%), Forever Living Products (16.3%), medicinal tea (14.4%), and black stone (12.5%). Prayer/faith healing (39.4%) was the second most common CAM after herbs. The alternative medical systems, such as energy techniques, manipulation, and body-based methods, were rarely used. Other CAMs used by small number of patients included ginger (1.9%), garlic (1.9%), *Noni *(1.9), mineral therapy (1.9%), bloodletting (1.9%), local surgery/scarifications (1.9%), divinations/incantations (1%), *qamwood *oil (*okwuma*) (1%), urine therapy (1%), magnetic water (1%), tuja 1000 (1%), *uda *(1%), mind-body technique (1%), manual healing/touch (1%) and green tea (1%). In particular, nutri water, kosagog tea, shark cartilage, animal extracts, hypnosis, psychic therapy, mental imagery, acupuncture, Indian medicine, chiropractics, bone setters/osteopathy, bioelectrical magnetism and oxygen/ozone therapy were never used by our patients.

**Table 2:** Types of CAM used by patients

**Biological products**	**Frequency of use (%)**	**Mind- body systems**	**Frequency (%)**
Herbs	54 (51.9)	Faith healing/prayer	40 (39.4)
Aloe vera	24 (23.1)	Visualization	9 (8.7)
Forever living products	17 (16.3)	meditation	7 (6.7)
Medicinal tea	15 (14.4)		
Python fat	8 (7.7)	**Alternative systems**	**Frequency**
Special diet/Nutritional therapy	7 (6.7)	Chinese medicine	9 (8.7)
High dose vitamins	5 (4.8)	Homeopathy	4 (3.8)
GNLD product	4 (3.8)	ritual sacrifice	3 (2.9)
*Tianshi*	3 (2.9)		
		**Others**	**Frequency**
		Black stone	13 (12.5)
		massage	4 (3.8)
		Mustard seed	3 (2.9)

Most of the respondents (77.9%) used CAM daily; others use them weekly (1.9%) or occasionally (6.7%), while 5.8% used them only once. The number of CAMs used by each patient varied from one to nine different types. The majority of the patients (61.5%) have used two or more types, 33.7% have used only one type, while 4.8% could not pick out the exact ones they used during this cancer. The majority of the patients (39.9%) have been on CAM for less than a month, 15.3% have used it for one to six months, 13.7% have used it for more than 6 months to one year, and 10.8% have used it for more than one year. Half of those using CAM (50.0%) have visited CAM practitioners several times during the course of this illness, 32.7% visited CAM practitioners once, and 7.7% have not visited any practitioner even though they were using CAM.

Most of the patients expected CAM to directly treat/cure their cancer (63.5%). Other expectations (Table 3) include: "just to do anything that will help fight the cancer" (17.3%); to improve physical well being (10.7%); to improve psychological/emotional well being (4.9%); relief of symptoms of cancer (2.9%); to improve body's ability to fight the cancer (1.9%). Most of the patients did not observe any benefit from CAM (67.3%), 4.8% of the respondents noticed some benefit they could not specify, 25% thought they experienced some specific benefits. Specific benefits reported include relief of pain (8 patients), feels healthy/good/improved/refreshes my body (5), reduction in swelling (3), prayer sustains my life (2), constipation relieved (2), helps to build my immune system/protects my body from other illness (2), healed wound (1), stopped bleeding in urine (1), felt it washed off disease from my stomach (1), relieved fever (1), stopped pushing effect in rectum (1), python fat cleared keloid at operation site (1).

**Table 3:** Expected and perceived (actual) benefits from CAM

**Expected benefits**	**Frequency (%)**	**Actual benefits**	**Frequency (%)**
Directly treat/cure the cancer	66 (63.5)	No benefit	70 (67.3)
To do everything possible for this cancer	18 (17.3)	Yes, can't specify	5 (4.8)
Improve physical well being	11 (10.7)	Yes with specific benefits	26 (25.0)
Improve psychological and emotional well being	5 (4.9)	No response	3 (2.9)
Relieve cancer symptoms	3 (2.9)		
Boost body's ability to fight	2 (1.9)		
Clean up wound	1 (1.0)		
Detect the type of disease and treat	1 (1.0)		
No response	6 (5.8)		
Total	104 (100)		

The majority of the patients (63.4%) did not experience any perceived unwanted effects from CAM; 5.8% reported unwanted effects but were not specific, while 22 patients (21.2%) reported various specific unwanted effects that include slimming down (3 patients), anorexia, nausea and vomiting (3 patients), weakness, malaise, generalized body discomfort (3 patients), and diarrhea/mucoid stool (3 patients). Other unwanted reactions included bleeding from wound and urine (2 patients), cough (2 patients), hotness of the body (2 patients), worsened stomach upset (2 patients), skin excoriations from balm (1 patient), constipation with frequent urination (1 patient), and lightheadedness (1 patient). While twenty-four patients (23.1%) were either very satisfied (1.9%) or satisfied (21.2%) with CAM, 68.3% said they were disappointed with it. Most patients (79.8%) said they would not repeat CAM or recommend it to someone they know for a similar condition, but 16.3 would recommend it. The majority of patients did not express an opinion (43.3%) or did not wish (38.5%) to include any aspect of CAM in orthodox medical practice. Among those who wanted some aspects of CAM to be used in conventional medicine (17.3%), the popular wishes were: Forever living products – 5.8%, prayers – 4.0%, and aloe vera – 2.9%.

The majority of CAM users (49.0%) did not have supervisors or guides for the CAM, but 41.3% had supervisors or someone guiding them. While 86.5% of the patients will continue to use orthodox medicine instead of CAM for this cancer, 9.6% said they will use the two together to help each other. Five respondents said that they had at one time or another abandoned conventional treatment in favor of CAM because they believed that CAM would cure them. About a third (32.7%) of CAM users discussed it with their doctors while 55.8% did not mention the CAM they were using to their doctors. Most CAM users (55.8%) declined to give reasons for their failure to mention the CAM to their doctors. The most common reasons given by those who answered the question were that the doctor did not ask (28.3%), or no specific reason (11.6%), or they felt the doctor would be unhappy, scold them, or tell them to stop (2.9%).

The respondents' main sources of information on CAM were family members (30.8%), friends (30.8%), and CAM practitioners (22.1%). Most patients obtained their CAM supply from the CAM practitioner (55.9%). Other significant sources included relations (13.5%), churches (10.6%), friends (8.7%), and the open market (7.7%).

## Discussion

To the best of our knowledge, this is the first study of the use of CAM therapies by cancer patients in any indigenous African population. The prevalence of CAM use among all cancer patients varies widely, even between reports on the same population. Figures have ranged from 7% to 83% [5,6], but the average rate across adult studies has been 31.4% [5]. Our own prevalence rate of 65.0% is one of the highest reported in the literature. While the definition of what is considered CAM therapy in each study accounts for some of these variations, we believe that the high prevalence rate among our patients can be explained by the traditional nature of our society, our cultural and religious beliefs and practices, the cost of western conventional treatment and our peoples' understanding of cancer as a disease. Nigeria is a developing country, predominantly traditional in outlook with 51.7% of the population living in rural areas and 57% of the people surviving on less than 1 dollar per day [16]. The current emphases on western biomedicine notwithstanding, traditional medical practices are still the mainstream ways to treat diseases and ailments for many Nigerians. Some individuals actually regard western biomedicine as an alternative or a complement to traditional medicine. While the two systems run parallel in the Nigerian environment, switching from one form to the other is a common phenomenon and depends on which of them each patient and the relations believe is most suitable for a particular condition. Also western biomedicine is expensive and relatively not accessible, compared to traditional forms of treatment. It is, therefore, not surprising that most patients resort to herbs and spiritual healing, upon which they have relied since antiquity. Many patients still regard cancer as due to spiritual forces beyond the reach of biomedicine. In addition, most of our cancer patients die even after receiving conventional western medical treatment. This gives people the impression that conventional treatments are not better. It therefore makes sense for them to try "alternative treatments" first, or to use them in combination with western biomedicine to get all the benefits possible. Studies from some other traditional societies such as Turkey and Taiwan documented similar high prevalence rates [11-13].

Conflicting findings have been reported about factors that affect the use of CAM therapies. Some studies have found associations between age, gender, socioeconomic status, income, and level of education. Studies in developed western countries found that women, in both the general population and among cancer patients, have higher prevalence of use than men, and that the peak age of use is among young adults/middle aged [3,10]. Also, higher income, higher level of education, and higher socioeconomic status have been linked to higher prevalence of CAM use [3,10]. Among a general population of Indians in Chatsworth, South Africa, Singh, et al. found no demographic factor to be a significant predictor of use of CAM [8]. Malik, et al. reported no significant influence of age, level of education, and socioeconomic status in a Turkish population of cancer patients, but found that patients from large families and women were more likely to use CAM [12]. Ceylan, et al. in a more recent survey in Turkey found no association between the use of CAM and age, gender, or marital status among cancer patients; they did, however, report an inverse relationship between use of CAM and socioeconomic status [11]. While we found a non-significant tendency for use of CAM to decrease with increasing levels of education, there was no association between use of CAM and age, marital status, or socioeconomic status in our patients. More of our women were non-CAM users. This finding stands out from all reports in the literature. We theorize that this is due to a cultural tendency of males from our population (as opposed to our females) to avoid western hospitals and try all forms of traditional therapy before resorting to conventional treatment.

CAM use in most developed countries in which health insurance is operational seems to be more among the better educated and higher socioeconomic classes since patients have to pay for the CAM privately. This trend does not exist in most poor developing countries where CAM is cheap and health insurance is nonexistent. Poor patients in developing countries who must pay out of their pocket for either CAM or the more expensive conventional treatment are more likely to try CAM first. It is also noteworthy that most studies from the developed world tend to be skewed towards the middle and higher income classes, and under-represent the lower socioeconomic class [3]. The demographics of the lower class however, are more likely to resemble the situation in resource-poor countries with respect to the use of CAM.

The most commonly used form of CAM in Nigeria is herbal preparations, followed by faith healing/prayer house healing. Singh, et al. recorded that herbs and spiritual healing were the two most common forms of CAM used among Indians in South Africa [9]. In the US, relaxation techniques, herbal medicine, massage, and chiropractics were the most commonly used forms of CAM within the general population [2]. Among cancer patients, spiritual practices, vitamins and herbs, movement and physical therapies, and mind/body approaches were most commonly used [8,17]. Herbal preparations have also been reported to be the leading CAM used among cancer patients in Turkey [11,12]. In the UK, the most common forms were healing, relaxation, and visualization [18]. Traditional African communities have always used herbs to cure diseases. Thus, it is not surprising that this practice has persisted and even extended to include newer preparations of biological products, such as aloe vera products, Forever living products, GNLD (Golden Neo life Diamite) products, medicinal tea etc.

The high prevalence of faith and prayer house healing among Nigerian patients mirrors the attachment of people of African ancestry to spiritual and transcendental beliefs. Some of the patients rely completely on prayers and faith for their healing. Such patients are usually brought to the hospital when the disease is widely metastatic or locally advanced. Deaths in such cases are inevitable, but also reinforce the belief that Western biomedicine is not helpful in treating cancer. Religious affiliations appear to influence the pattern of use of CAM in our population, but not at statistically significant levels. More of the Pentecostals in our population used CAM compared to other groups and this is accounted for mainly by use of faith/prayer house healing. The high proportion of Catholics among our study population explains the increased prevalence in the use of religious relics and items such as black stone, olive oil, and mustard seed.

Most of our patients expected CAM to directly treat or cure their cancers (63.5%). Similar findings were observed among cancer patients in the U.K [18], and Turkey [11]. In contrast, cancer patients in the US use CAM primarily to improve quality of life, boost the immune system, and relieve symptoms with only one-third of patients expecting a direct curative treatment from the CAM [8]. The high rate of disappointment (68.3%) that our patients expressed about the performance of CAM in cancer is explained by the fact that most of them were hoping for a direct treatment/cure; it also explains their reluctance (79.8%) to recommend it to someone else or use it in the future for cancer treatment. Indeed, the fact that only 2.9% of users among our patients got information about their CAM from other patients supports the data that most of them were disappointed and will not recommend it or use it in future. CAM users in Nigeria rely on family members, friends, and CAM practitioners for their information about CAM. Surprisingly, none of our patient said that they received information on CAM from the mass media – despite the fact that CAM practitioners often use it as a means to promote their products.

Up to 25% of our respondents claimed they got some specific benefits from CAM; almost an equal number (23.1%) were either very satisfied or satisfied with the results. Some of the benefits remain subjective and unverifiable – for example: "it makes me feel healthy"; "it helps my body fight cancer"; "it sustains my life"; "it cleanses my body." While some of the observed benefits, such as relief of pain and reduction in size of lumps, can be verified, the fact that CAM is used in conjunction with conventional treatment casts doubts on these claims.

Only 21.2% of respondents reported unwanted effects from CAM, supporting the assumption that these agents are natural and safe. Most of the claimed adverse effects are very difficult to distinguish from the natural manifestations or progression of advanced malignancies. Some specific adverse effects are however indisputable. Over the course of this study, we observed two cases of full thickness chemical burns following application of herbs on the skin. CAM's potential for serious harm is evidenced by the fact that five of our patients abandoned conventional treatment in favor of CAM. While most CAM may not be directly injurious to the patient, the greatest harm may be in delaying or preventing patients from coming for potentially curative cancer treatment at the earliest possible time.

Most of our patients who used CAM (55.8%) did not tell their doctors about it – mainly because the doctor failed to ask (28.3%). This finding is in keeping with reported disclosure rates of 39%–45.8% in studies of cancer patients in the US [4], UK [18] and Indians in South Africa [11]. The fact that most patients will not disclose their use of CAM unless asked makes it necessary for every oncologist to routinely ask cancer patients whether they use CAM, the ones they use, and how they use them. A small proportion of our patients did not inform their doctors because they feared that the doctor would scold them or tell them to stop it. This finding underscores the necessity for oncologists to refrain from being judgmental about patients' use of CAM if they are to learn the truth.

The main weakness of our study is that it is a hospital-based survey, thereby excluding patients who have abandoned conventional treatment completely or never used it at all. Also our questionnaire was developed after review of existing studies, most of which were from industrialized western countries. While we made it relevant to our local environment by incorporating local CAMs and local ideas on CAM, there is still the possibility that some valuable insights on the Nigerian patients' attitudes to biomedicine and traditional medical practices (regarded as CAM by biomedicine) might not be conveyed by the method we used. We believe such insights may be better gained through qualitative in-depth individual and focus group interviews. Also, our patient population does not represent all cancers in the hospital. Hematological malignancies, gynecological malignancies, and some subspecialty areas were under-represented because these patients are not managed within the core surgical oncology sections in our hospital. These shortcomings notwithstanding, we believe the study gives a reliable picture of the use of CAM among cancer patients in a teaching hospital in Nigeria.

## Conclusion

The prevalence of CAM use in cancer patients in Nigeria is one of the highest in the world. While it tends to be less common in females, it is not affected by age, marital status, socioeconomic status, or level of education. Herbs and faith healing/prayer house healing are the most common forms of CAM. Most of the patients expect to be cured and are disappointed with the results of CAM. A majority of the patients who used CAM did not volunteer that information to their doctors, primarily because the doctors did not ask about it. We believe that every clinical oncologist should be aware of the prevalence of use of CAM in his/her environment. The physician should find out the composition of the various therapies and know which ones are harmful to patients. Patients should routinely be asked about CAM and its use as part of every cancer patient's evaluation.

## Competing interests

We have no competing financial or non-financial interests in this study except to advance knowledge.

## Authors' contributions

ERE conceived this study, participated in the design, data collection, analysis, and writing. ANE participated in the design, data collection, and writing of the article. Both authors read and approved the final manuscript.

## Pre-publication history

The pre-publication history for this paper can be accessed here:



## Supplementary Material

Additional file 1 Questionnaire on the use of complementary and alternative medicine by cancer patients at the University of Nigeria Teaching Hospital, Enugu, Nigeria. This final validated questionnaire incorporated local CAMS and relevant issues on the use of CAM in a developing country like Nigeria. Click here for file
